# Chemotherapy delivery time affects treatment outcomes of female patients with diffuse large B cell lymphoma

**DOI:** 10.1172/jci.insight.164767

**Published:** 2023-01-24

**Authors:** Dae Wook Kim, Ja Min Byun, Jeong-Ok Lee, Jae Kyoung Kim, Youngil Koh

**Affiliations:** 1 Department of Mathematical Sciences, KAIST, Daejeon, South Korea.; 2 Biomedical Mathematics Group, Institute for Basic Science, Daejeon, South Korea.; 3 Department of Internal Medicine, Seoul National University College of Medicine, Seoul National University Hospital, Seoul, South Korea.; 4 Department of Internal Medicine, Seoul National University College of Medicine, Seoul National University Bundang Hospital, Seongnam, South Korea.

**Keywords:** Hematology, Oncology, Cancer, Cancer immunotherapy, Lymphomas

## Abstract

**BACKGROUND:**

Chronotherapy is a drug intervention at specific times of the day to optimize efficacy and minimize adverse effects. Its value in hematologic malignancy remains to be explored, in particular in adult patients.

**METHODS:**

We performed chronotherapeutic analysis using 2 cohorts of patients with diffuse large B cell lymphoma (DLBCL) undergoing chemotherapy with a dichotomized schedule (morning or afternoon). The effect of a morning or afternoon schedule of rituximab plus cyclophosphamide, doxorubicin, vincristine, and prednisone (R-CHOP) on survival and drug tolerability was evaluated in a survival cohort (*n* = 210) and an adverse event cohort (*n* = 129), respectively. Analysis of about 14,000 healthy individuals followed to identify the circadian variation in hematologic parameters.

**RESULTS:**

Both progression-free survival (PFS) and overall survival (OS) of female, but not male, patients were significantly shorter when patients received chemotherapy mostly in the morning (PFS HR 0.357, *P* = 0.033; and OS HR 0.141, *P* = 0.032). The dose intensity was reduced in female patients treated in the morning (cyclophosphamide 10%, *P* = 0.002; doxorubicin 8%, *P* = 0.002; and rituximab 7%, *P* = 0.003). This was mainly attributable to infection and neutropenic fever: female patients treated in the morning had a higher incidence of infections (16.7% vs. 2.4%) and febrile neutropenia (20.8% vs. 9.8%) as compared with those treated in the afternoon. The sex-specific chronotherapeutic effects can be explained by the larger daily fluctuation of circulating leukocytes and neutrophils in female than in male patients.

**CONCLUSION:**

In female DLBCL patients, R-CHOP treatment in the afternoon can reduce toxicity while it improves efficacy and survival outcome.

**FUNDING:**

National Research Foundation of Korea (NRF) grant funded by the Korean government (grant number NRF-2021R1A4A2001553), Institute for Basic Science IBS-R029-C3, and Human Frontiers Science Program Organization Grant RGY0063/2017.

## Graphical abstract

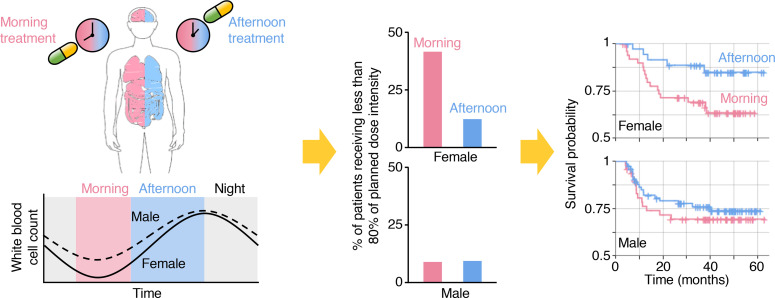

## Introduction

Although cancer survival has dramatically increased over the past 20 years, cancer remains one of the leading causes of death worldwide (1). To address this, the major focus has traditionally been on developing novel anticancer drugs (2). However, more physicians are recognizing the importance of optimal usage of existing drugs. Among such efforts, there has been a longstanding interest in the strategy of treating cancer according to biological rhythms, i.e., chronotherapy (3).

Specifically, more than 3 decades ago, it was shown that patients with ovarian cancer less frequently experienced dose delays and reductions if treatment with doxorubicin and cisplatin were given in the morning and the evening, respectively (4). Indeed, during the past 30 years, several experimental and clinical studies have demonstrated positive associations between the circadian clock and drug responses in patients with cancer (5–8). In particular, nearly 50 anticancer drugs displayed chronotoxicity (9, 10), indicating timed treatment can increase dose levels without sacrificing the toxicity. The most solid evidence is available for colorectal cancers, with randomized trial data supporting the chronomodulated delivery of fluorouracil, leucovorin, and oxaliplatin (10). However, the exciting findings in colorectal cancers have failed to alter routine clinical practice because of a lack of consistency in subsequent trials (6, 11–13). Recent series of meta-analyses revealed that such discrepancies in colorectal cancers could be due to the sex moderation of the chronotherapeutic effect (14–16). This notion is further supported by emerging evidence of sexual dimorphism in the circadian systems (17–22). Interestingly, like cytotoxic drugs, timed immune checkpoint inhibitor treatment of melanoma and non–small cell lung cancer also significantly improves survival outcomes (23, 24), implying the importance of the chronotherapy in immunotherapy.

On the other hand, the effect of chronotherapy on hematologic malignancy treatment outcomes requires further exploration. Only sparse data are available from children with acute lymphoblastic leukemia (25), and the value of treatment timing in adult patients with hematologic malignancies remains unclear. As such, we performed this study to explore the feasibility of implementing chronomodulated immunochemotherapy for diffuse large B cell lymphoma (DLBCL) treatment. We chose DLBCL for the following reasons: (i) since rituximab plus cyclophosphamide, doxorubicin, vincristine, and prednisone (R-CHOP) is the sole treatment option for most DLBCL cases, confounding factors are negligible and the treatment outcomes almost entirely depend on the efficacy and toxicity of the immunochemotherapy; (ii) about 30% of patients with DLBCL ultimately experience refractory/relapse disease, and so far myriads of attempts to improve survival outcomes have not yielded strongly positive results (26–28), thus leaving room for better therapeutic tactics (29); (iii) evidence exists for circadian variation in the tolerability and antitumor efficacy of cyclophosphamide (30–33), doxorubicin (30, 33, 34), and vincristine (10) in human or animal studies; and (iv) there is room for chronotherapeutic effect in immunotherapy with rituximab, and thus it should be investigated (23). Moreover, all chemotherapy treatments start at either 8:30 AM (morning) or 2:30 PM (afternoon) at our centers’ chemotherapy day units. Such binary timing of chemotherapy delivery sets the stage for investigating the clinical relevance of chronotherapy.

## Results

### Baseline characteristics of DLBCL cohorts.

**Figure 1:**
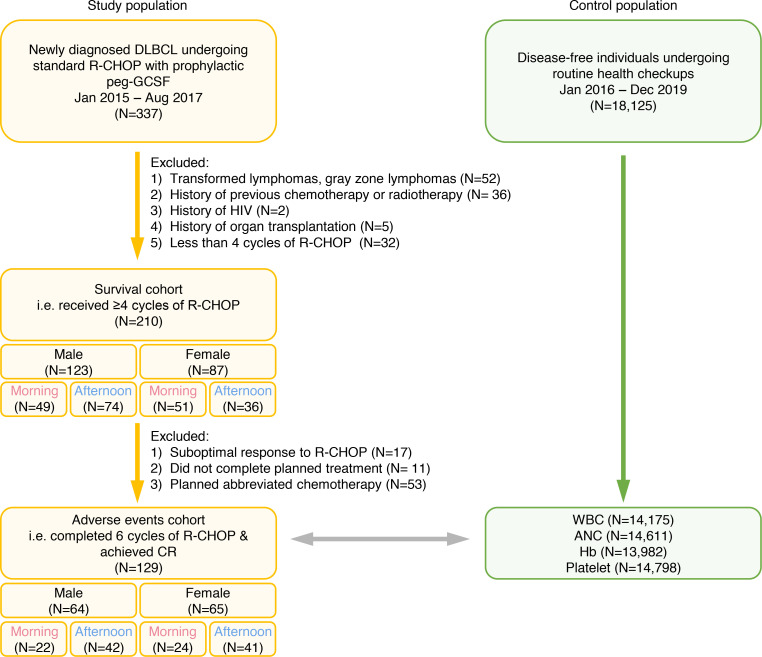
CONSORT diagram describing the flow of patient enrollment. Peg, pegylated; CR, complete remission; ANC, absolute neutrophil count; Hb, hemoglobin.

This study consists of 2 cohorts: a survival cohort and an adverse event cohort as shown in the CONSORT diagram (Figure 1) (see Methods for details). The baseline characteristics of the survival cohort are shown in Table 1 and those of the adverse events cohort in Table 2. There was no difference in age at diagnosis, stage, ECOG performance status, and R-IPI scores between both treatment timing groups. The median age at DLBCL diagnosis was 61 years (range 27–77), and there were no differences across the groups. All patients were classified as relatively fit with ECOG performance < 2. About half of the patients had stage 3–4 disease, and the composition was similar across the groups. Most patients had an R-IPI score of 2 or 3; no patients had an R-IPI score of 5.

### Survival cohort analysis.

During the median follow-up of 40.7 months (IQR 23.5–50.7 months), the median progression-free survival (PFS) was not reached, and the median overall survival (OS) was 86 months for the entire cohort. Most patients achieved complete remission at completion of chemotherapy (Table 1).

**Figure 2:**
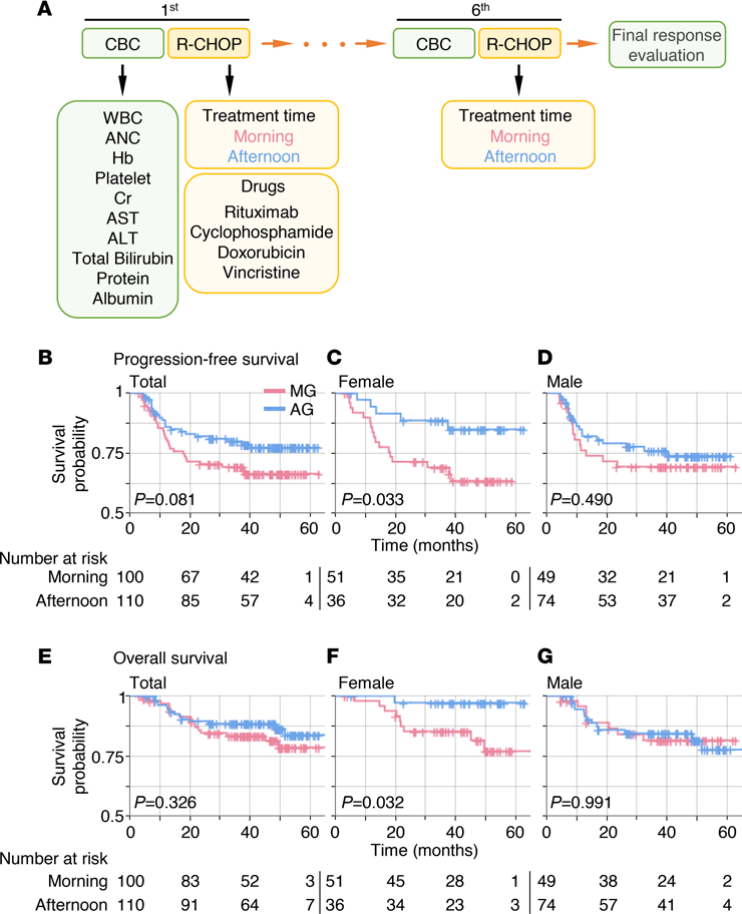
Kaplan-Meier curves of PFS and OS according to the time of the day of R-CHOP delivery and sex. (**A**) The procedure of R-CHOP chemotherapy. (**B**–**G**) PFS of MG and AG (**B**), female MG and AG (**C**), and male MG and AG (**D**) and OS of MG and AG (**E**), female MG and AG (**F**), and male MG and AG (**G**). Cross (+) indicates censoring of data. Here, 210 patients in the survival cohort were analyzed. *P* values were calculated by log-rank test.

**Figure 3:**
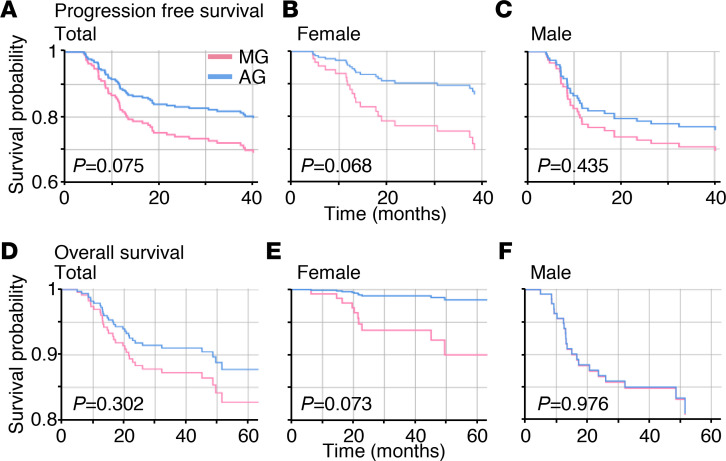
Even after adjusting for R-IPI, PFS and OS decrease more rapidly in the female MG than in the female AG. (**A**–**F**) Cox proportional-hazard survival curves of PFS (**A**–**C**) and OS (**D**–**F**) according to sex and time of the day of R-CHOP delivery after adjusting for R-IPI (Supplemental Figure 1). PFS of MG and AG (**A**), female MG and AG (**B**), and male MG and AG (**C**) and OS of MG and AG (**D**), female MG and AG (**E**), and male MG and AG (**F**). PFS (**A**) and OS (**D**) are not different between MG and AG. However, PFS (**B**) and OS (**E**) are different between the female MG and AG: they decrease more rapidly in the female MG than in the female AG. In contrast, there is no difference between the male MG and AG (**C** and **F**). Here, 210 patients in the survival cohort were analyzed. *P* values were calculated by Wald’s test.

For female patients, the morning group (MG) experienced more frequent disease progression compared with the afternoon group (AG) (33.3% vs. 13.9%, *P* = 0.040). This translated into significantly shorter PFS for the female MG (median not reached for both groups; HR 0.357; *P* = 0.033; Figure 2C). Even after R-IPI adjustment (Supplemental Figure 1), the female MG showed a tendency for shorter PFS compared with the female AG (HR 0.393; *P* = 0.068; Figure 3B). In contrast, no difference was seen in the male patients (Figure 2D).

Likewise, more deaths were noted in the female MG compared with the female AG (19.6% vs. 2.8%, *P* = 0.020). The most common cause of death was disease progression. Consequently, the female MG showed shorter OS (median 86 months, 3-year OS rate 69.2%) compared with the female AG (median not reached, 3-year OS rate 88.6%) (HR 0.141; *P* = 0.032; Figure 2F). After R-IPI adjustment (Supplemental Figure 1; supplemental material available online with this article; https://doi.org/10.1172/jci.insight.164767DS1), the female MG showed a tendency for shorter OS compared with the female AG (HR 0.150; *P* = 0.073; Figure 3E). Chemotherapy delivery in the morning (*P* = 0.043), chemotherapy delivery at older age (*P* = 0.013), and chemotherapy delivery in stage 3–4 disease (*P* = 0.015) were recognized as poor prognostic factors for OS by multivariate analysis (Table 3). OS was not affected by the chemotherapy delivery time for male patients (Figure 2G). Risk factors regarding PFS and OS are shown in more detail using forest plots in Supplemental Figure 2.

### Adverse event cohort analysis.

**Figure 4:**
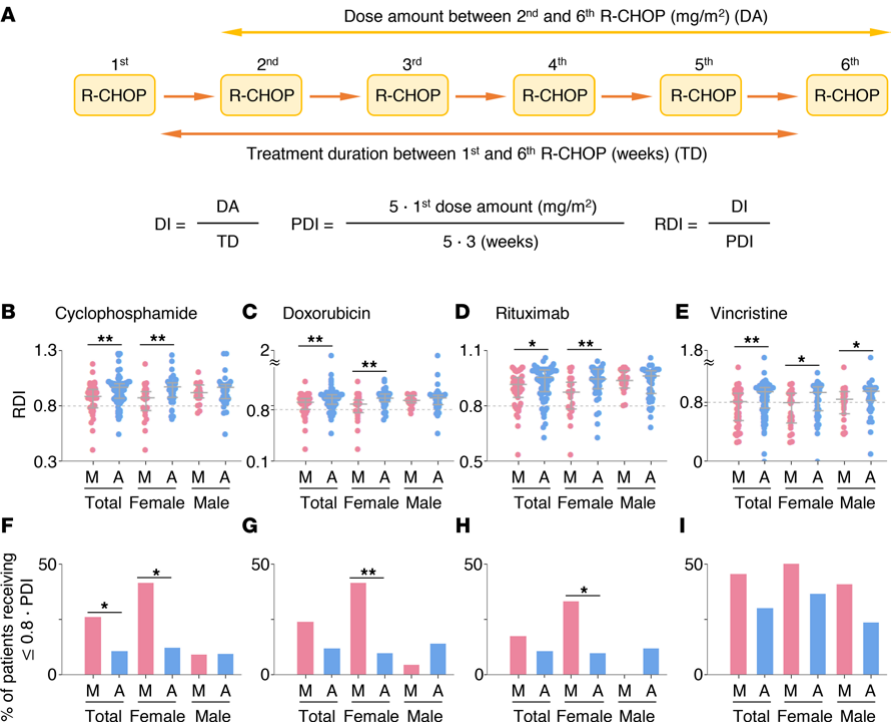
Dose intensity of cyclophosphamide, doxorubicin, rituximab, and vincristine according to sex and the time of the day of R-CHOP delivery. (**A**) The definition of dose intensity (DI), planned dose intensity (PDI), and relative dose intensity (RDI). (**B**–**E**) RDI of cyclophosphamide (**B**), doxorubicin (**C**), rituximab (**D**), and vincristine (**E**) in MG and AG, female MG and AG, and male MG and AG. (**F**–**I**) The fraction of patients receiving less than 80% of PDI of cyclophosphamide (**F**), doxorubicin (**G**), rituximab (**H**), and vincristine (**I**) in MG and AG, female MG and AG, and male MG and AG. Here, 129 patients in the adverse event cohort were analyzed. *P* values in **B**–**E** and those in **F**–**I** were calculated by Mann-Whitney *U* test and χ^2^ test, respectively. Error bar denotes IQR. *P < 0.05; **P < 0.01.

There were no differences in the baseline WBC, ANC, hemoglobin, and platelet counts among the groups (Table 2). However, the female MG experienced more dose delays compared with the female AG (33.3% vs. 9.8%; *P* = 0.042; Table 2). Moreover, RDI decreased in female patients when they received R-CHOP in the morning (Figure 4, A–E). Thus, the proportion of patients receiving less than 80% of planned dose intensity was higher in the female MG compared with the female AG (Figure 4, F–H). In contrast, there was no statistically significant difference between the male MG and the male AG, in particular for cyclophosphamide, doxorubicin, and rituximab (Table 2 and Figure 4). Importantly, this sex-dependent pattern in doxorubicin was observed commonly even when we separately analyzed the 2 independent subcohorts making up the adverse event cohort (Supplemental Figure 3).

We also explored how RDI changes during the time course of the treatment according to sex and the time of the day of R-CHOP by calculating the RDI for each treatment cycle (Supplemental Figure 4A). Specifically, if the accumulated number of morning treatments until the *n*th treatment is equal to or larger than that of the accumulated number of afternoon treatments, we subgrouped patients into the *n*th accumulated morning group (Supplemental Figure 4, B–I). Otherwise, they were subgrouped into the *n*th accumulated afternoon group. We found that when the treatment was more often administered in the morning than in the afternoon, the RDI of cyclophosphamide, doxorubicin, and rituximab decreased in female patients while the RDI did not change in male patients (Supplemental Figure 4, B–G). Such a decrease of RDI in the female accumulated morning group was still observed even when patients were subgrouped for each treatment cycle (Supplemental Figure 5).

The most common reason for cyclophosphamide dose reduction was infection/neutropenic fever (*n* = 6). Among these patients, 5 also underwent concurrent doxorubicin reduction. Three patients experienced doxorubicin reduction because of compromised cardiac function. Decrease in rituximab RDI was mostly due to dose delays, but 2 patients experienced absolute dose reduction due to rituximab-related pneumonitis.

The female MG experienced more incidences of febrile neutropenia (20.8% vs. 9.8%) and viral infections (16.7% vs. 2.4%) compared with the female AG, with the latter showing statistical significance (*P* = 0.038, Table 2).

### Hematologic parameter analysis in reference cohort.

**Figure 5:**
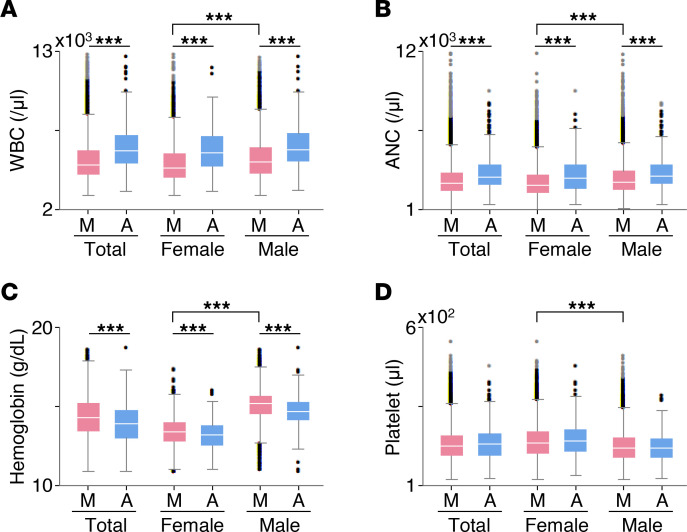
WBC, ANC, Hb, and platelet abundance according to sex and time of the day of measurement. Here, box-and-whisker graphs describing the distribution of the hematology parameters were plotted. The outliers that are defined to be outside 1.5 times IQR either above the upper quartile or below the lower quartile are denoted by circles. Lines within boxes represent medians. The sample numbers of WBC (**A**), ANC (**B**), Hb (**C**), and platelet (**D**) abundance are 6,447, 6,720, 6,736, and 6,882 in the female group and 7,728, 7,891, 7,246, and 7,916 in the male group, respectively. *P* values were calculated by Mann-Whitney *U* test. ***P < 0.001.

To investigate the possible role of diurnal variation of hematologic parameters on the compromised treatment schedule and dose, we constructed a reference cohort to serve as the control population. We reviewed WBC, ANC, hemoglobin, and platelet counts of disease-free individuals according to sex and time of the day of measurement (Figure 5). Specifically, patients whose hematologic parameters were measured between 8 am and 12 pm and between 2 pm and 6 pm were subgrouped into the MG and the AG, respectively. We found clear diurnal variations in WBC and ANC (Figure 5, A and B), which is consistent with previous studies (35–37). Fold-change of diurnal variations in WBC were 1.22 in female (*P* = 7.57 × 10^–23^) and 1.16 in male (*P* = 2.44 × 10^–21^) (Figure 5A). Furthermore, WBC and ANC were lowest in the female MG (Figure 5, A and B) (median WBC 4.9 × 10^3^/μL female MG vs. 5.3 × 10^3^/μL male MG, *P* = 6.8 × 10^–66^; and median ANC 2.7 × 10^3^/μL female MG vs. 2.9 × 10^3^/μL male MG, *P* = 5.9 × 10^–42^).

## Discussion

Despite the establishment of R-CHOP as the standard frontline therapy of DLBCL with success in improving survival outcomes, about one-third of patients ultimately experience relapse (38, 39). Various strategies to improve R-CHOP efficacy have been explored, including dose intensification, substitution with newer anti-CD20 agents, and addition of new agents to the R-CHOP backbone (3). In our study, we explored the role of chronotherapy for optimal delivery of R-CHOP. The importance of our study lies in suggesting that (i) the timing of chemoimmunotherapy delivery can affect the treatment outcomes in relation to maximal tolerated dose; (ii) a chronotherapeutic approach influences women and men differently; and (iii) these differences are reflected by underlying diurnal variations in serum biochemical and hematological measurements.

One of the most prominent findings of our study is that female patients with DLBCL receiving R-CHOP in the morning more often experienced significant reduction of DI leading to compromised survival (Figures 2–4) mainly due to infection and neutropenic fever (Table 2). Notably, the trend in DI was overall preserved in the 2 independent subcohorts (Supplemental Figure 3), indicating that our findings are not due to a hidden bias in retrospective studies. Importantly, these findings match the observation from recent randomized clinical studies that female patients with colorectal cancer receiving irinotecan in the afternoon experienced fewer toxicities (15, 16). These support our results and provide a possibility to generalize them to other cancers. Specifically, it has been reported that irinotecan delivered to female patients with colorectal cancer in the morning more often led to neutropenia (15). Validating our results, we also found solid evidence of naturally occurring diurnal leukopenia/neutropenia, which may be the culprit leading to the sex moderation of the chronotherapeutic effect (Figure 5). It is plausible that these sexual differences in WBC/ANC diurnal variations might be related to the previous findings for colorectal cancer (10, 15, 16) because irinotecan is a topoisomerase 1 inhibitor whose properties are similar to those of intercalating agents such as doxorubicin (10).

In this study, the survival outcomes of male patients with DLBCL were not statistically different between the MG and the AG. However, PFS of men seemed to be better in the AG than in the MG as observed in female patients (Figure 2D), indicating that the optimal timing of R-CHOP for male patients may be similar to that of female patients. Such subtle improvement from the afternoon treatment in male patients compared with female patients might be attributable to the smaller diurnal fluctuation in hemograms in males (Figure 5) (35). Another possibility is that the optimal timing for male patients occurs at times that were not investigated in our study (e.g., in the middle of the night). Indeed, the least toxic timing of irinotecan for male patients with colorectal cancer is about 6 hours earlier than that of female patients (15, 40). It would be interesting to explore if the time points not considered in this study might affect the outcomes of male patients with DLBCL .

The chronotherapeutic impact of R-CHOP indicates the potential chronotherapeutic impact on immunotherapy. Indeed, recent retrospective clinical studies found that timed immune checkpoint inhibitor infusion can increase survival in patients with melanoma (23) and patients with non–small cell lung cancer (24). The major anticancer mechanisms of rituximab in DLBCL are antibody-dependent cellular cytotoxicity and complement-dependent cytotoxicity, both of which are closely related to immunity (41). Furthermore, rituximab affects hemograms and generates infection susceptibility (42). As a result, cytotoxic agents (cyclophosphamide, vincristine, and doxorubicin) and a immunotherapeutic agent (rituximab) can have poor tolerability when administered in the morning. Although efforts to reach beyond R-CHOP to maximize DLBCL treatment outcomes have led to recognition of DI as an important prognostic factor, the exact methodology of optimal dose intensification is yet to be determined. For example, the RICOVER-60 trial comparing 6 versus 8 cycles of R-CHOP14 failed to show survival gain with more cycles of R-CHOP (43). Subsequent phase III trials comparing frontline R-CHOP14 versus standard R-CHOP (28, 44) also failed to show survival benefits. Taking our findings into context, rather than uniformly increasing the chemotherapy dose for all, chronomodulated R-CHOP delivery for women may guide us toward effective yet safe dose intensification.

Several previous studies provide robust evidence for sex-specific chronotherapeutic effects (14–19, 45). Specifically, timed immune checkpoint inhibitor treatment improves clinical outcomes in melanoma for female patients more than male patients (23), possibly because women are less susceptible to circadian disruption of the immune system than men (46, 47). In the field of hematologic malignancies, Scheving et al. reported that the cure rate of female leukemic mice treated with cyclophosphamide was lower when the drug was given at night than during the daytime, and the opposite pattern was observed in male leukemic mice (32). This finding is in line with our results because nocturnal mice manifest inverse circadian rhythms compared with humans (48). The concept of sex-oriented dose intensification is not new in DLBCL: current National Comprehensive Cancer Network guidelines suggest 500 mg/m^2^ rituximab is more efficacious than the conventional 375 mg/m^2^ for elderly male patients (49). It can be surmised that for successful dose intensification of R-CHOP, both sex and the timing of the chemotherapy delivery should be considered.

Although we suggest a biologic mechanism for hematologic toxicity based on the larger fluctuation of hemograms in women compared with men, a more detailed mechanism for this phenomenon should be delineated in future studies. In fact, previous studies also revealed circadian fluctuation of both hemogram and DNA synthetic activity of bone marrow (37, 50–52). These studies consistently showed peak immune cell count in the afternoon or evening as in our findings (Figure 5). As there should be a time lag between bone marrow DNA synthesis and peripheral blood myeloid cell appearance, considering the time for maturation of innate immune cells including monocytes and neutrophils, morning appears to be the time when proliferative activity of bone marrow is high (50–52). This high proliferative activity of bone marrow in the morning and the large daily fluctuation in females would constitute a biological background for worse outcomes in female patients receiving R-CHOP chemotherapy in the morning.

Cancers show temporal variation in their propagation that is controlled by the circadian clock (10). For example, non-Hodgkin’s lymphomas show circadian variation in cell cycle distribution, potentially resulting in temporal variation in cancer propagation (53). Similarly, the sexual dimorphism in chronotherapeutic effect might be associated with the circadian clock from a molecular viewpoint. Specifically, it might be related to the core clock molecules CLOCK and REV-ERBα because they have a sex-specific genotype or phenotype (19, 31, 54, 55). As CLOCK is known to be associated with the lymphocyte survival/recovery rate (31), investigating its link with the sex-specific chronotherapeutic effect in hematologic malignancy would be interesting for future work.

In this study, patient subgrouping is based on external clock time (i.e., 8:30 am or 2:30 pm) as done in previous chronotherapy studies (6, 16, 23, 56). This might not accurately capture the patients’ internal circadian time (57): individuals with the same sleep-wake cycle can have different internal circadian times by up to 5 hours (58). To circumvent this limitation, the patients’ individual circadian time needs to be noninvasively measured in free-living conditions. Recently, several noninvasive internal circadian time detection methods have been proposed (57, 59–64). It would be important in future work to study the chronotherapeutic effect with the internal circadian time that can be estimated by these methods. Another issue to be addressed is the imbalance between groups on confounding factors that possibly affect the outcome of the analysis. Fortunately, as shown in Tables 1 and 2, there were no significant differences between the 2 groups in our study. However, there is a imbalanced sex ratio between the MG and the AG in the survival cohort: the number of female patients is 51 in the MG of 100 patients and 36 in the AG of 110 patients. This might be associated with social factors, such as men being more economically active than women in South Korea. Due to morning work schedules, economically active populations often prefer afternoon or evening sessions, leading to an allocation of more female patients to the morning session than to the afternoon session. Better designed prospective trials should ensue to address these unresolved issues. Last, although there was a graphically appreciable decrease in survival probability when female patients mostly underwent R-CHOP even after adjusting for R-IPI, the statistical significance decreased (Figures 2 and 3). This is possibly attributable to the relatively low number of patients in this study, because this can allow the similarity of the survival curves in the early period (e.g., <10 months) to largely reduce the statistical significance. Larger scaled studies should ensue to address this problem.

All in all, we explored the possibility of implementing sex-oriented chronotherapy as an alternative axis of augmenting DLBCL frontline therapy. We conclude that female patients should, if possible, avoid receiving R-CHOP in the morning for optimal chemotherapy delivery with maximum response.

## Methods

### Study design and participants.

This was a longitudinal cohort study of newly diagnosed DLBCL patients more than 18 years old treated with first-line R-CHOP at Seoul National University Hospital and Seoul National University Bundang Hospital daytime chemotherapy centers between January 2015 and August 2017. As mentioned, the chemotherapy centers at each hospital are open for chemotherapy twice a day. Patients undergoing chemotherapy predominantly at 8:30 am were classified as the MG, while those undergoing chemotherapy predominantly at 2:30 pm were classified as the AG.

As shown in the CONSORT diagram (Figure 1), this study was constructed in 2 parts. The first part focused on the relationship between differences in daily treatment timing and survival outcomes (i.e., survival cohort). Initially, 337 patients were identified. After excluding patients with transformed lymphomas or gray zone lymphoma (*n* = 52), a history of previous chemotherapy or radiotherapy (*n* = 36), a history of human immunodeficiency virus infection (*n* = 2), or a history of organ transplantation (*n* = 5) and those receiving fewer than 4 cycles of R-CHOP (*n* = 32), a total of 210 patients were deemed eligible. Patients undergoing fewer than 4 cycles of R-CHOP were omitted from the survival cohort analyses to minimize bias, as these patients represent a predefined favorable outcomes group (65). Their medical records were reviewed for demographics, disease characteristics, response to treatment, and survival outcomes.

The second part of the study focused on determining the effects of chemotherapy delivery timing on toxicity and DI (i.e., adverse event cohort). For this part, we analyzed the adverse events and laboratory findings of the 129 patients in the survival cohort who completed 6 cycles of R-CHOP and achieved complete remission at the end of the treatment (Figure 1). Specifically, out of 210 patients, 146 patients underwent 6 cycles of R-CHOP. Of these, only 129 achieved complete remission, as seen in Figure 1. We chose this group as our adverse event cohort to minimize other confounding factors and concentrate solely on the causal relationship between chemotherapy and adverse events.

A control population of disease-free individuals undergoing routine health checkups between January 2016 and December 2019 at Seoul National University Hospital was appointed to provide data on normal diurnal variations of laboratory findings. A blinded set of laboratory results on WBC (reference range 3.0 × 10^9^/L to 11.0 × 10^9^/L), ANC (reference range 1.0 × 10^9^/L to 8.0 × 10^9^/L), hemoglobin (reference range 11.0 g/dL to 18.0 g/dL), and platelet count (reference range 100 × 10^9^/L to 500 × 10^9^/L) was extracted and used. The parameters were collected per event, not per person. At the end, 14,175 WBC counts, 14,611 ANC counts, 13,982 hemoglobin counts, and 14,798 platelet counts were taken into account to construct a reference cohort.

### DI calculation and response and toxicity evaluation of R-CHOP.

A full-dose cycle of R-CHOP consisted of i.v. rituximab 375 mg/m^2^, cyclophosphamide 750 mg/m^2^, doxorubicin 50 mg/m^2^, vincristine 1.4 mg/m^2^ on day 1, and oral prednisone 100 mg from day 1 to 5, repeated at 21-day intervals. Total treatment duration between the first R-CHOP and the sixth R-CHOP was 115 days on average (IQR 107–126 days) in the survival cohort and 112 days on average (IQR 106–120 days) in the adverse event cohort. Pegylated GCSF was used in all patients. For each patient, the DI of cyclophosphamide, doxorubicin, rituximab, and vincristine was calculated. Specifically, to entirely focus on the dependence of DI on the treatment time, DI was calculated by dividing the total dose amount between the second and the sixth treatments by the number of weeks of the entire treatment duration. The RDI was calculated as the ratio of DI to planned DI as defined in previous work (26). The incidence of chemotherapy dose delays, dose reductions, and treatment discontinuations was also analyzed. A dose delay was defined as a >5-day delay for any subsequent cycle after cycle 1. A dose reduction was defined as 20% reduction from the planned standard dose for cyclophosphamide, doxorubicin, vincristine, and rituximab during any cycle.

Tumor response assessment for R-CHOP was performed according to the Lugano response criteria (66). PFS was defined as the time from chemotherapy administration to disease progression or death from any cause. OS was defined as the time from chemotherapy administration to death from any cause. The adverse events were assessed according to the National Cancer Institute Common Terminology Criteria for Adverse Events version 4.03 (67).

### Statistics.

PFS was calculated from the date of diagnosis to the date of relapse, progression, or death or of the last follow-up. OS was calculated from the date of diagnosis to the date of death or to the date of the last follow-up. The survival functions of PFS and OS were estimated with the Kaplan-Meier method and compared by log-rank test. The Cox proportional-hazard model was used to estimate the survival functions of PFS and OS after adjusting for the R-IPI, and the estimated functions were compared by Wald’s test. The association between potential prognostic factors and survival outcomes was evaluated using Cox’s proportional-hazard regression model with a stepwise backward variable selection procedure. Specifically, if the predictors had a *P* value greater than 0.05, they were removed. As a result, only the predictors that had a *P* value of less than 0.05 were considered in the regression models.

Baseline characteristics of the patient population are described as percentages (Tables 1 and 2). Continuous variables that passed the Shapiro-Wilk normality test are provided as mean (SD) or mean (SEM) as specified. Continuous variables that did not pass the normality test are provided as median (IQR). Bivariate comparisons were performed using the 2-tailed *t* test for normally distributed continuous variables in Table 1 and Table 2 or the Mann-Whitney *U* test for continuous variables not normally distributed. Bivariate comparisons of categorical variables (i.e., the percentage of patients with the respective attribute, such as sex) were done with the χ^2^ test. All data were analyzed using IBM SPSS Statistics, version 22.0. *P* values of less than 0.05 were considered statistically significant.

### Study approval.

This study was conducted according to the Declaration of Helsinki and was approved by the Institutional Review Board of Seoul National University Hospital (IRB No. H-1910-015-106, Seoul, South Korea). The need for informed consent was waived in light of the retrospective nature of the study and the anonymity of the participants.

## Author contributions

YK and JKK were responsible for designing the review protocol, writing the article, screening potentially eligible studies, and interpreting data. DWK was responsible for analyzing data, interpreting data, updating the reference list, creating figures, and writing the article. JMB was responsible for analyzing data, interpreting data, and writing the article. JMB and JOL contributed to data extraction.

## Supplementary Material

Supplemental data

ICMJE disclosure forms

## Figures and Tables

**Table 1:**
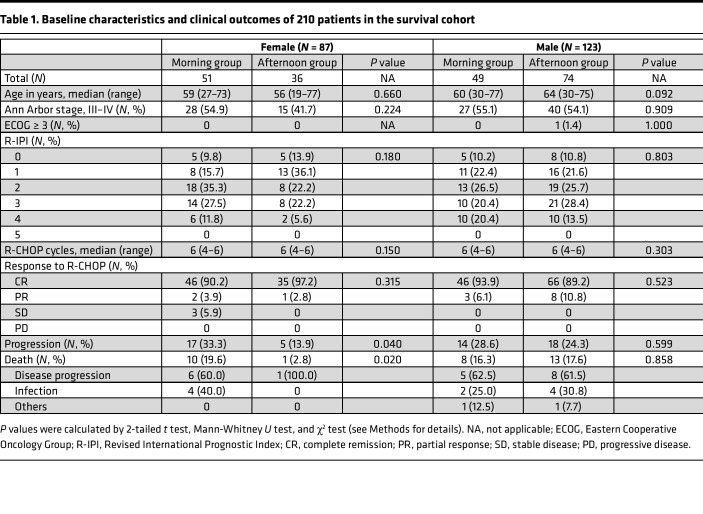
Baseline characteristics and clinical outcomes of 210 patients in the survival cohort

**Table 2:**
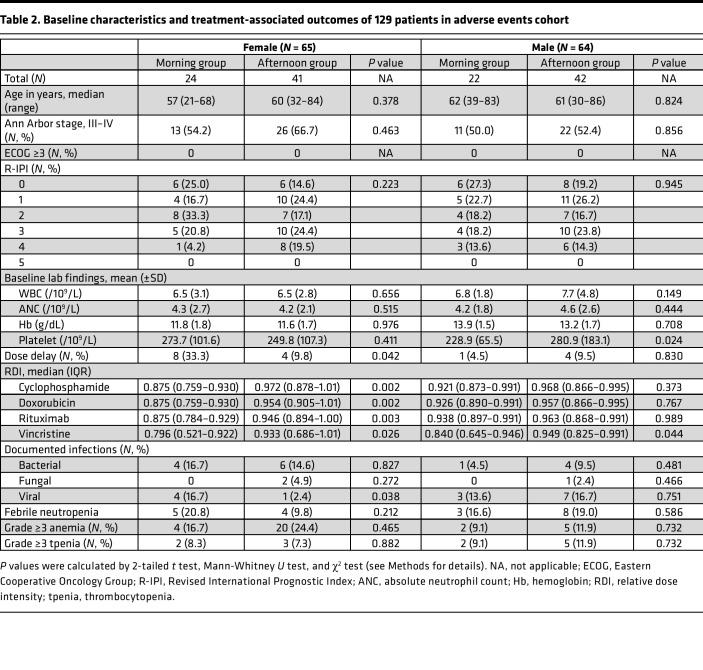
Baseline characteristics and treatment-associated outcomes of 129 patients in adverse events cohort

**Table 3:**
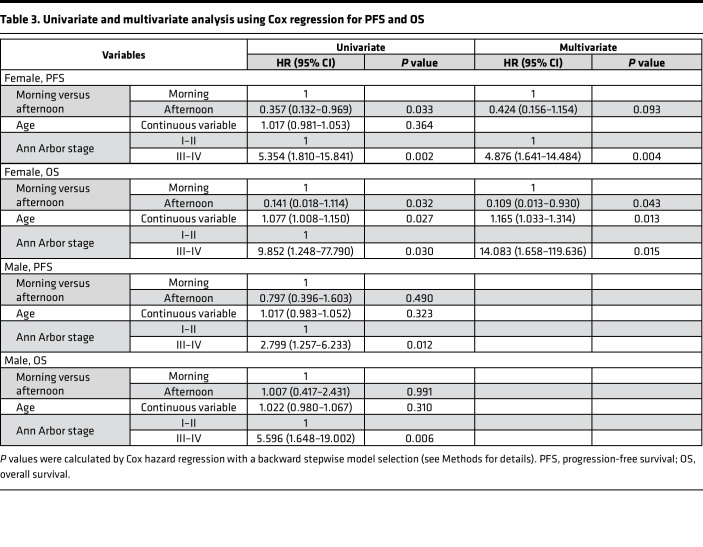
Univariate and multivariate analysis using Cox regression for PFS and OS

## Version 1 12/13/2022

In-Press Preview

## Version 2 01/24/2023

Electronic publication
