# Supplementary material for: Chemotherapy delivery time affects treatment outcomes of female patients with diffuse large B cell lymphoma
Source: JCI Insight. 2023 Jan 24;8(2):e164767. doi: 10.1172/jci.insight.164767 (PMC9977288; doi:10.1172/jci.insight.164767)
Supplement: Supplemental data [file jciinsight-8-164767-s090.pdf]

Supplemental Figures for

Chemotherapy delivery time affects treatment outcomes of female patients with diffuse large B-cell lymphoma

Dae Wook Kim[1,2,5,a], Ja Min Byun[3,5], Jeong-Ok Lee[4], Jae Kyoung Kim[1,2*], Youngil Koh[3*]

[1]Department of Mathematical Sciences, Korea Advanced Institute of Science and Technology, Daejeon, Korea, 34141

[2]Biomedical Mathematics Group, Institute for Basic Science, Daejeon, Korea, 34126

[3]Department of Internal Medicine, Seoul National University College of Medicine, Seoul National University Hospital, Seoul, Korea, 110744

[4]Department of Internal Medicine, Seoul National University College of Medicine, Seoul National University Bundang Hospital, Seongnam, Korea, 463707

[5]Equally contributed

[a]Current Address: Department of Mathematics, University of Michigan, Ann Arbor, MI, USA, 48109

*Corresponding authors. Email: go01@snu.ac.kr and jaekkim@kaist.ac.kr

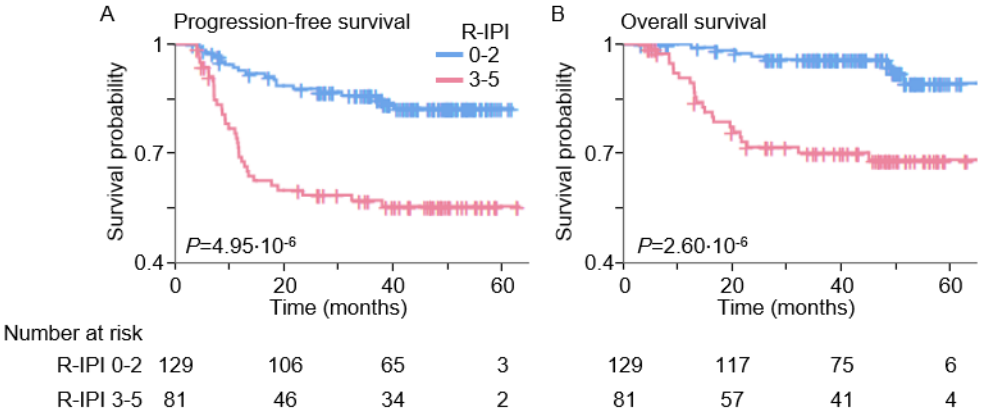

Supplemental Figure 1. Kaplan-Meier curves of progression-free survival (A) and overall survival (B) according to R-IPI. PFS and OS decreased in patients with R-IPI 0-2 compared to patients with R-IPI 3-5. Cross (+) indicates censoring of data. Here, 129 patients with R-IPI 0-2 and 81 patients with R-IPI 3-5 in the survival cohort were analyzed. *P*-values were calculated by log-rank test.

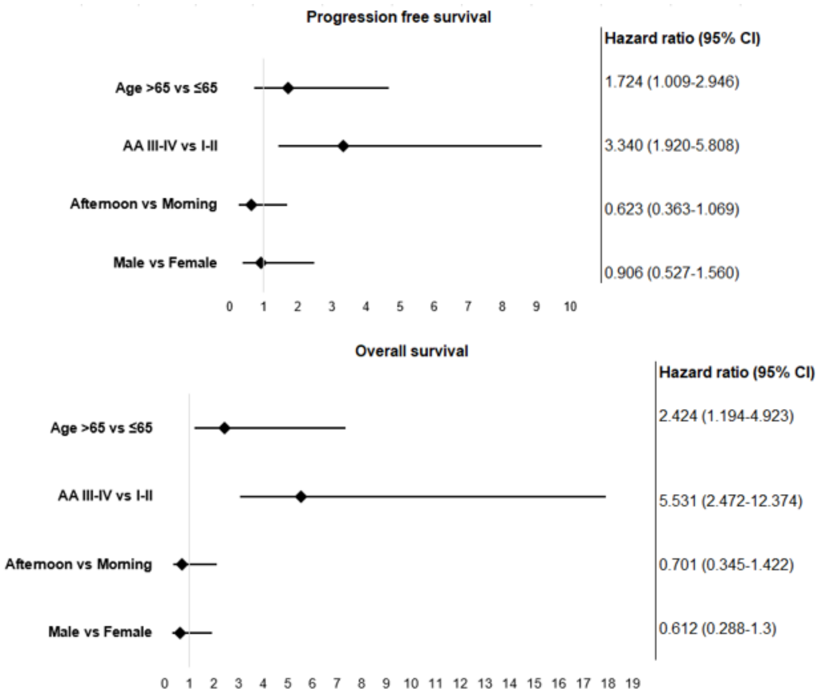

Supplemental Figure 2. Forest plots showing hazard ratios for survival. The upper and lower panels show the hazard ratios for progression-free survival and overall survival, respectively. Here, 210 patients in the survival cohort were analyzed.

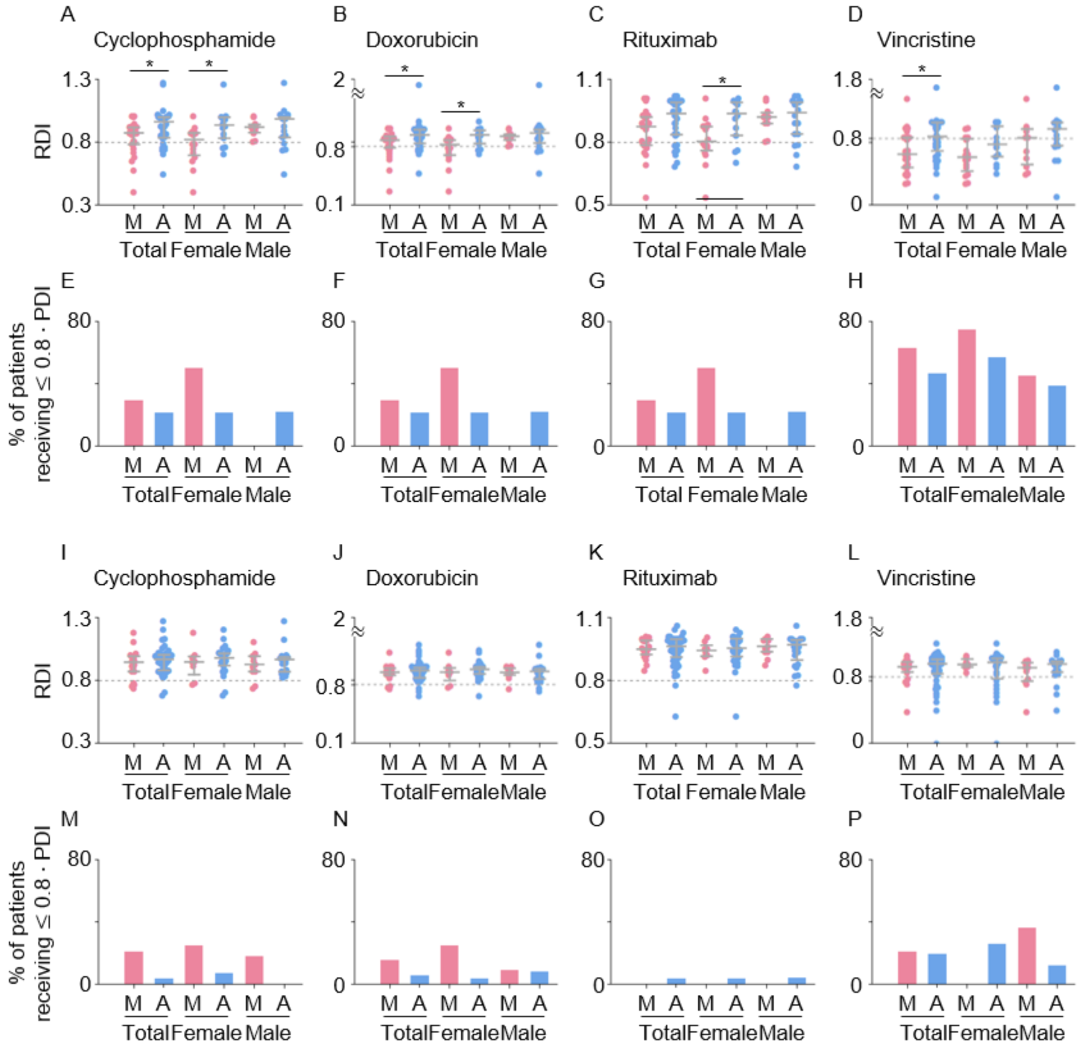

Supplemental Figure 3. Dose intensity according to sex and the time of the day of R-CHOP delivery in the two independent cohorts. (A-D) RDI (relative dose intensity) of cyclophosphamide (A), doxorubicin (B), rituximab (C), and vincristine (D) in MG and AG, female MG and AG, and male MG and AG of the Seoul National University Hospital adverse event cohort. **(E-H)** The fraction of patients receiving less than 80% of PDI (planned dose intensity) of cyclophosphamide (E), doxorubicin (F), rituximab (G), and vincristine (H) in MG and AG, female MG and AG, and male MG and AG of the Seoul National University Hospital adverse event cohort. **(I-L)** RDI of cyclophosphamide (I), doxorubicin (J), rituximab (K), and vincristine (L) in MG and AG, female MG and AG, and male MG and AG of the Seoul National

University Bundang Hospital adverse event cohort. **(M-P)** The fraction of patients receiving less than 80% of PDI of cyclophosphamide (M), doxorubicin (N), rituximab (O), and vincristine (P) in MG and AG, female MG and AG, and male MG and AG of the Seoul National University Bundang Hospital adverse event cohort. Here, RDI was calculated as in Figure 4. 129 patients in the adverse event cohort were analyzed. *P*-values in (A-D) and (I-L) were calculated by Mann-Whitney *U* test. *P*-values in (E-H) and (M-P) were calculated by Chi-square test. Error bar denotes IQR.

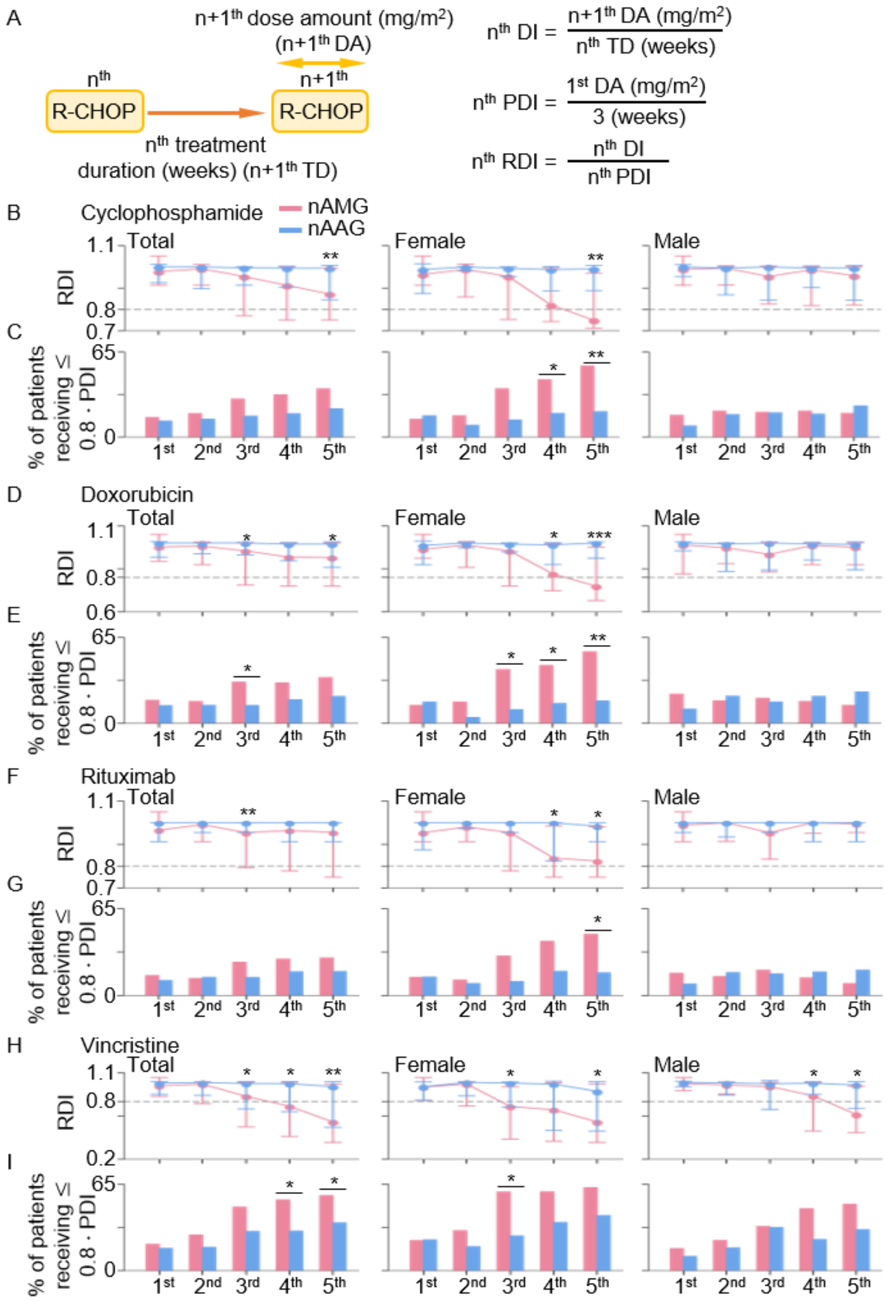

Supplemental Figure 4. Dose intensity for a treatment cycle according to sex and the accumulated number of morning treatments before the cycle. (A) Definition of nth DI (dose intensity), nth PDI (planned dose intensity), and nth RDI (relative dose intensity). **(B-I)** nth RDI and the fraction of patients receiving less than 80% of nth PDI of cyclophosphamide (B and C), doxorubicin (D and E), rituximab (F and G), and vincristine (H and I) for each cycle. Here,

when analyzing the nth RDI, patients are subgrouped into the nth accumulated morning group (nAMG) if the number of morning treatments is equal to or larger than that of afternoon treatments until the nth treatment. Otherwise, they are subgrouped into the nth accumulated afternoon group (nAAG). RDI decreases in the nAMG but not in the nAAG when treatment is ongoing (B, D, F, and H left). Thus, RDI becomes lower in the nAMG than in the nAAG for the latter part of the R-CHOP chemotherapy (i.e., 3rd, 4th and 5th therapies). In particular, it becomes statistically significant in the last (i.e., 5th) measured RDI except for rituximab. Such lower RDI of cyclophosphamide, doxorubicin, and rituximab in the nAMG than in the nAAG is mainly due to the lower RDI in the female nAMG than in the female nAAG in the latter part of the therapy (B, D, and F center); RDI in male patients is not different between the nAMG and the nAAG (B, D, and F right). Accordingly, the fraction in the latter part becomes higher in the female nAMG than in the female nAAG (C, E, and G center) while there is no difference between the male nAMG and the male nAAG (C, E, and G right). Unlike other drugs, RDI of vincristine decreases in both the female nAMG and the male nAMG (H). However, the fraction in the latter is not statistically different between the nAMG and the nAAG in either female or male patients (I). Here, 129 patients in the adverse event cohort were analyzed. *P*-values in (B, D, F, and H) and those in (C, E, G, and I) were calculated by Mann-Whitney *U* test and Chi-square test, respectively. Solid circles and error bars in B, D, F, and H denote median and IQR.

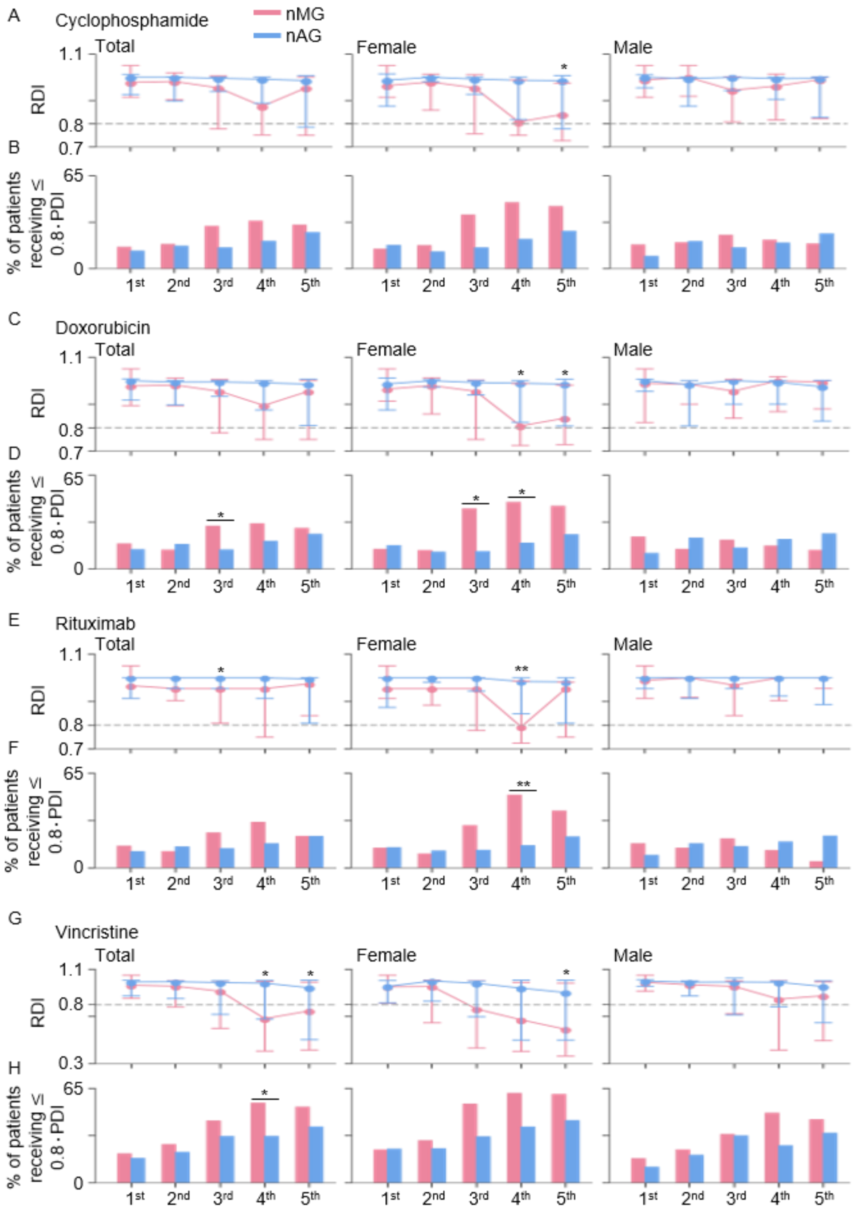

Supplemental Figure 5. Dose intensity for a specific treatment cycle according to sex and time of the day of R-CHOP before the cycle. (A-H) nth RDI and the fraction of patients receiving less than 80% of nth PDI of cyclophosphamide (A and B), doxorubicin (C and D), rituximab (E and F), and vincristine (G and H) for each cycle. Here, when analyzing the nth RDI, patients are subgrouped into the nth morning group (nMG) if their previous treatment time is morning. Otherwise, they are subgrouped into the nth afternoon group (nAG). When

treatment is in progress, RDI of cyclophosphamide, doxorubicin, and rituximab decreases in the nMG, in particular after the 4th treatment, while it does not in the nAG (A, C, E, and G left). This is mainly due to the lower RDI in the female nMG than in the female nAG in the later treatments (A, C, and E center); there is no difference between the male nMG and the male nAG (A, C, and E right). Accordingly, there is a higher fraction in the female nMG receiving less than 80% of PDI than in the female nAG; the majority show a very slight trend toward significance ($P < 0.2$) in the latter parts of the therapy (B, D, and F center). In contrast, there is no such trend for the male patients (B, D, and F right). The RDI of vincristine decreases in both the female nAMG and the male nAMG (G). However, the fraction receiving less than 80% of PDI in the later treatments is not statistically different between the nAMG and the nAAG in either female or male patients (H). Here, 129 patients in the adverse event cohort were analyzed. *P*-values in (A, C, E, and G) and those in (B, D, F, and H) were calculated by Mann-Whitney *U* test and Chi-square test, respectively. Solid circles and error bars in A, C, E, and G denote median and IQR.
