# Supplementary material for: Chemotherapy delivery time affects treatment outcomes of female patients with diffuse large B cell lymphoma
Source: JCI Insight. 2023 Jan 24;8(2):e164767. doi: 10.1172/jci.insight.164767 (PMC9977288; doi:10.1172/jci.insight.164767)
Supplement: ICMJE disclosure forms [file jciinsight-8-164767-s091.pdf]

ICMJE DISCLOSURE FORM

**Date:**	11/1/2022
**Your Name:**	Youngil Koh
**Manuscript Title:**	Chemotherapy delivery time affects treatment outcome of female patients with diffuse large B-cell lymphoma
**Manuscript Number (if known):**	Click or tap here to enter text.

In the interest of transparency, we ask you to disclose all relationships/activities/interests listed below that are related to the content of your manuscript. "Related" means any relation with for-profit or not-for-profit third parties whose interests may be affected by the content of the manuscript. Disclosure represents a commitment to transparency and does not necessarily indicate a bias. If you are in doubt about whether to list a relationship/activity/interest, it is preferable that you do so.

The author's relationships/activities/interests should be defined broadly. For example, if your manuscript pertains to the epidemiology of hypertension, you should declare all relationships with manufacturers of antihypertensive medication, even if that medication is not mentioned in the manuscript.

In item #1 below, report all support for the work reported in this manuscript without time limit. For all other items, the time frame for disclosure is the past 36 months.

		**Name all entities with whom you have this relationship or indicate none (add rows as needed)**	**Specifications/Comments (e.g., if payments were made to you or to your institution)**
		**Time frame: Since the initial planning of the work**	
1	All support for the present manuscript (e.g., funding, provision of study materials, medical writing, article processing charges, etc.) **No time limit for this item.**	☐ None	
		Korean Government (MSIP)	NRF-2021R1A4A2001553
			Click the tab key to add additional rows.
		**Time frame: past 36 months**	
**2**	Grants or contracts from any entity (if not indicated in item #1 above).	☐ **None**	
		Korean Government (MSIP)	
		Korean Government (MOHW)	
		Sanofi Genzyme	
**3**	Royalties or licenses	☐ **None**	

		Name all entities with whom you have this relationship or indicate none (add rows as needed)	Specifications/Comments (e.g., if payments were made to you or to your institution)
**4**	Consulting fees	☐ **None**	
		MSD	
		Janssen	
		Novartis	
		Takeda	
**5**	Payment or honoraria for lectures, presentations, speakers bureaus, manuscript writing or educational events	☐ **None**	
		Amgen	
		Janssen	
		BMS	
		Novartis	
		Pfizer	
		MSD	
**6**	Payment for expert testimony	☒ **None**	
**7**	Support for attending meetings and/or travel	☒ **None**	
**8**	Patents planned, issued or pending	☒ **None**	
**9**	Participation on a Data Safety Monitoring Board or Advisory Board	☐ **None**	
		Xencor	
		Prothena	
**10**	Leadership or fiduciary role in other board, society, committee or advocacy group, paid or unpaid	☒ **None**	

		Name all entities with whom you have this relationship or indicate none (add rows as needed)	Specifications/Comments (e.g., if payments were made to you or to your institution)
**11**	Stock or stock options	☐ **None**	
		GenomeOpinion	
		DeepMetrics	
		Proteina	
		Curocell	
		Tomocube	
**12**	Receipt of equipment, materials, drugs, medical writing, gifts or other services	☒ **None**	
**13**	Other financial or non-financial interests	☒ **None**	

Please place an "X" next to the following statement to indicate your agreement:

☒ I certify that I have answered every question and have not altered the wording of any of the questions on this form.

ICMJE DISCLOSURE FORM

Date: 11/2/2022

Your Name: Jae Kyoung Kim

Manuscript Title: Chemotherapy delivery time affects treatment outcome of female patients with diffuse large B-cell lymphoma

Manuscript Number (if known): Click or tap here to enter text.

In the interest of transparency, we ask you to disclose all relationships/activities/interests listed below that are related to the content of your manuscript. "Related" means any relation with for-profit or not-for-profit third parties whose interests may be affected by the content of the manuscript. Disclosure represents a commitment to transparency and does not necessarily indicate a bias. If you are in doubt about whether to list a relationship/activity/interest, it is preferable that you do so.

The author's relationships/activities/interests should be defined broadly. For example, if your manuscript pertains to the epidemiology of hypertension, you should declare all relationships with manufacturers of antihypertensive medication, even if that medication is not mentioned in the manuscript.

In item #1 below, report all support for the work reported in this manuscript without time limit. For all other items, the time frame for disclosure is the past 36 months.

		**Name all entities with whom you have this relationship or indicate none (add rows as needed)**	**Specifications/Comments (e.g., if payments were made to you or to your institution)**
**Time frame: Since the initial planning of the work**			
1	All support for the present manuscript (e.g., funding, provision of study materials, medical writing, article processing charges, etc.) **No time limit for this item.**	☐ None	
		Life Science Editors	
			Click the tab key to add additional rows.
**Time frame: past 36 months**			
**2**	Grants or contracts from any entity (if not indicated in item #1 above).	☐ **None**	
		Institute for Basic Science	IBS-R029-C3
		Human Frontiers Science Program Organization Grant	RGY0063/2017
**3**	Royalties or licenses	☒ **None**	

		Name all entities with whom you have this relationship or indicate none (add rows as needed)	Specifications/Comments (e.g., if payments were made to you or to your institution)
**4**	Consulting fees	☒ **None**	
**5**	Payment or honoraria for lectures, presentations, speakers bureaus, manuscript writing or educational events	☒ **None**	
**6**	Payment for expert testimony	☒ **None**	
**7**	Support for attending meetings and/or travel	☒ **None**	
**8**	Patents planned, issued or pending	☒ **None**	
**9**	Participation on a Data Safety Monitoring Board or Advisory Board	☒ **None**	
**10**	Leadership or fiduciary role in other board, society, committee or advocacy group, paid or unpaid	☒ **None**	

		Name all entities with whom you have this relationship or indicate none (add rows as needed)	Specifications/Comments (e.g., if payments were made to you or to your institution)
**11**	Stock or stock options	☒ **None**	
**12**	Receipt of equipment, materials, drugs, medical writing, gifts or other services	☒ **None**	
**13**	Other financial or non-financial interests	☒ **None**	

Please place an "X" next to the following statement to indicate your agreement:

☒ I certify that I have answered every question and have not altered the wording of any of the questions on this form.

ICMJE DISCLOSURE FORM

**Date:**	11/2/2022
**Your Name:**	Dae Wook Kim
**Manuscript Title:**	Chemotherapy delivery time affects treatment outcome of female patients with diffuse large B-cell lymphoma
**Manuscript Number (if known):**	Click or tap here to enter text.

In the interest of transparency, we ask you to disclose all relationships/activities/interests listed below that are related to the content of your manuscript. "Related" means any relation with for-profit or not-for-profit third parties whose interests may be affected by the content of the manuscript. Disclosure represents a commitment to transparency and does not necessarily indicate a bias. If you are in doubt about whether to list a relationship/activity/interest, it is preferable that you do so.

The author's relationships/activities/interests should be defined broadly. For example, if your manuscript pertains to the epidemiology of hypertension, you should declare all relationships with manufacturers of antihypertensive medication, even if that medication is not mentioned in the manuscript.

In item #1 below, report all support for the work reported in this manuscript without time limit. For all other items, the time frame for disclosure is the past 36 months.

		**Name all entities with whom you have this relationship or indicate none (add rows as needed)**	**Specifications/Comments (e.g., if payments were made to you or to your institution)**
**Time frame: Since the initial planning of the work**			
1	All support for the present manuscript (e.g., funding, provision of study materials, medical writing, article processing charges, etc.) **No time limit for this item.**	☒ None	
			Click the tab key to add additional rows.
**Time frame: past 36 months**			
**2**	Grants or contracts from any entity (if not indicated in item #1 above).	☒ **None**	
**3**	Royalties or licenses	☒ **None**	

		Name all entities with whom you have this relationship or indicate none (add rows as needed)	Specifications/Comments (e.g., if payments were made to you or to your institution)
**4**	Consulting fees	☐ **None**	
**5**	Payment or honoraria for lectures, presentations, speakers bureaus, manuscript writing or educational events	☒ **None**	
**6**	Payment for expert testimony	☒ **None**	
**7**	Support for attending meetings and/or travel	☒ **None**	
**8**	Patents planned, issued or pending	☒ **None**	
**9**	Participation on a Data Safety Monitoring Board or Advisory Board	☒ **None**	
**10**	Leadership or fiduciary role in other board, society, committee or advocacy group, paid or unpaid	☒ **None**	

		Name all entities with whom you have this relationship or indicate none (add rows as needed)	Specifications/Comments (e.g., if payments were made to you or to your institution)
**11**	Stock or stock options	☒ **None**	
**12**	Receipt of equipment, materials, drugs, medical writing, gifts or other services	☒ **None**	
**13**	Other financial or non-financial interests	☒ **None**	

Please place an "X" next to the following statement to indicate your agreement:

☒ I certify that I have answered every question and have not altered the wording of any of the questions on this form.

ICMJE DISCLOSURE FORM

**Date:**	11/2/2022
**Your Name:**	Ja Min Byun
**Manuscript Title:**	Chemotherapy delivery time affects treatment outcome of female patients with diffuse large B-cell lymphoma
**Manuscript Number (if known):**	Click or tap here to enter text.

In the interest of transparency, we ask you to disclose all relationships/activities/interests listed below that are related to the content of your manuscript. "Related" means any relation with for-profit or not-for-profit third parties whose interests may be affected by the content of the manuscript. Disclosure represents a commitment to transparency and does not necessarily indicate a bias. If you are in doubt about whether to list a relationship/activity/interest, it is preferable that you do so.

The author's relationships/activities/interests should be defined broadly. For example, if your manuscript pertains to the epidemiology of hypertension, you should declare all relationships with manufacturers of antihypertensive medication, even if that medication is not mentioned in the manuscript.

In item #1 below, report all support for the work reported in this manuscript without time limit. For all other items, the time frame for disclosure is the past 36 months.

		**Name all entities with whom you have this relationship or indicate none (add rows as needed)**	**Specifications/Comments (e.g., if payments were made to you or to your institution)**
**Time frame: Since the initial planning of the work**			
1	All support for the present manuscript (e.g., funding, provision of study materials, medical writing, article processing charges, etc.) **No time limit for this item.**	☒ None	
			Click the tab key to add additional rows.
**Time frame: past 36 months**			
**2**	Grants or contracts from any entity (if not indicated in item #1 above).	☒ **None**	
**3**	Royalties or licenses	☒ **None**	

		Name all entities with whom you have this relationship or indicate none (add rows as needed)	Specifications/Comments (e.g., if payments were made to you or to your institution)
**4**	Consulting fees	☒ **None**	
**5**	Payment or honoraria for lectures, presentations, speakers bureaus, manuscript writing or educational events	☒ **None**	
**6**	Payment for expert testimony	☒ **None**	
**7**	Support for attending meetings and/or travel	☒ **None**	
**8**	Patents planned, issued or pending	☒ **None**	
**9**	Participation on a Data Safety Monitoring Board or Advisory Board	☒ **None**	
**10**	Leadership or fiduciary role in other board, society, committee or advocacy group, paid or unpaid	☒ **None**	

		Name all entities with whom you have this relationship or indicate none (add rows as needed)	Specifications/Comments (e.g., if payments were made to you or to your institution)
**11**	Stock or stock options	☒ **None**	
**12**	Receipt of equipment, materials, drugs, medical writing, gifts or other services	☒ **None**	
**13**	Other financial or non-financial interests	☒ **None**	

Please place an "X" next to the following statement to indicate your agreement:

☒ I certify that I have answered every question and have not altered the wording of any of the questions on this form.

ICMJE DISCLOSURE FORM

Date: 11/2/2022

Your Name: Jeong-OK Lee

Manuscript Title: Chemotherapy delivery time affects treatment outcome of female patients with diffuse large B-cell lymphoma

Manuscript Number (if known): Click or tap here to enter text.

In the interest of transparency, we ask you to disclose all relationships/activities/interests listed below that are related to the content of your manuscript. "Related" means any relation with for-profit or not-for-profit third parties whose interests may be affected by the content of the manuscript. Disclosure represents a commitment to transparency and does not necessarily indicate a bias. If you are in doubt about whether to list a relationship/activity/interest, it is preferable that you do so.

The author's relationships/activities/interests should be defined broadly. For example, if your manuscript pertains to the epidemiology of hypertension, you should declare all relationships with manufacturers of antihypertensive medication, even if that medication is not mentioned in the manuscript.

In item #1 below, report all support for the work reported in this manuscript without time limit. For all other items, the time frame for disclosure is the past 36 months.

		**Name all entities with whom you have this relationship or indicate none (add rows as needed)**	**Specifications/Comments (e.g., if payments were made to you or to your institution)**
**Time frame: Since the initial planning of the work**			
1	All support for the present manuscript (e.g., funding, provision of study materials, medical writing, article processing charges, etc.) **No time limit for this item.**	☒ None	
			Click the tab key to add additional rows.
**Time frame: past 36 months**			
**2**	Grants or contracts from any entity (if not indicated in item #1 above).	☒ **None**	
**3**	Royalties or licenses	☒ **None**	

		Name all entities with whom you have this relationship or indicate none (add rows as needed)	Specifications/Comments (e.g., if payments were made to you or to your institution)
**4**	Consulting fees	☒ **None**	
**5**	Payment or honoraria for lectures, presentations, speakers bureaus, manuscript writing or educational events	☒ **None**	
**6**	Payment for expert testimony	☒ **None**	
**7**	Support for attending meetings and/or travel	☒ **None**	
**8**	Patents planned, issued or pending	☒ **None**	
**9**	Participation on a Data Safety Monitoring Board or Advisory Board	☒ **None**	
**10**	Leadership or fiduciary role in other board, society, committee or advocacy group, paid or unpaid	☒ **None**	

		Name all entities with whom you have this relationship or indicate none (add rows as needed)	Specifications/Comments (e.g., if payments were made to you or to your institution)
**11**	Stock or stock options	☒ **None**	
**12**	Receipt of equipment, materials, drugs, medical writing, gifts or other services	☒ **None**	
**13**	Other financial or non-financial interests	☒ **None**	

Please place an "X" next to the following statement to indicate your agreement:

☒ I certify that I have answered every question and have not altered the wording of any of the questions on this form.

JCI The Journal of Clinical Investigation

Click on boxes to check/uncheck items.

Checklist for submitting a revised Clinical Medicine manuscript

In addition to addressing the items noted in the decision letter regarding your manuscript, ensure that your revised manuscript adheres to the guidelines below. For full submission details, visit the JCI website.

Required files

Manuscript

☑ PDF of a clean version of the entire manuscript; include references, figures, figure legends, and tables.

☑ PDF of a marked-up version of the entire manuscript showing revisions and beginning with a point-by-point response to reviewer comments.

☑ Word or RTF file of all manuscript text; include references, figure legends, tables, and table legends (<u>but not</u> figures, images, markup, or point-by-point responses).

☑ Single PDF file of completed ICMJE uniform disclosure forms from all authors.

☐ For clinical trials, a PDF of the appropriate reporting checklist (CONSORT, STROBE. etc.).

Figures

☑ Publication-quality figures in TIFF format. See detailed instructions for figure preparation.

☑ Recommended: Graphical abstract (details available here).

Supplemental material

☑ Supplemental information, figures, and modest-sized tables, as:

- ☑ (a) (if applicable) a PDF in which reviewer-requested changes are highlighted
- ☑ (b) a clean, publication-quality PDF

☐ Upload any supplemental videos and/or large spreadsheets separately.

☑ Before submission, carefully review all supplemental files; they will not be checked by a copy editor. The Journal is not responsible for any errors contained in supplemental material.

Blot/gel images

☐ For any figure showing a cropped blot or gel: a PDF, PPT, or PPTX file (distinct from any other supplemental material) that shows the unedited blot or gel images in their entirety.

☐ Annotate each image as, e.g., "Full unedited gel for Figure 2B."

☐ Clearly indicate which bands were used for the figures.

☐ View Journal policy on gel/blot images.

Formatting and style

☑ Recommended 9,000/maximum 12,000 words (including title page, full text, references, figure legends, and tables).

☑ Double-spaced throughout, including references and tables; figure legends may be single spaced if necessary to keep a figure and its legend on the same page.

☑ All pages are numbered.

☑ Each section begins on a new page.

Abbreviations and acronyms

☑ Standard JCI abbreviations and acronyms are used without definition.

☑ All other abbreviations and acronyms are spelled out at first use in the Abstract and again at first use in the main text (with the abbreviated form appearing in parentheses) and used without definition thereafter.

Gene names and symbols

☐ Gene names and symbols conform to official NCBI Gene Nomenclature.

☐ Presented according to JCI Gene nomenclature and style.

Italicization

☐ Generally reserved for gene symbols, genotypes, and species names.

☐ Terms such as in vivo, in vitro, etc., are not italicized.

Unpublished data, manuscripts in preparation or under review, and personal communications

☑ Cited parenthetically in the text, not as numbered references; e.g., "(Jane L. Doe, UCLA, Los Angeles, California, USA, unpublished observations)."

☐ Written permission to cite unpublished observations by individuals external to the author's research team (email is sufficient) is submitted.

Reference citations

☑ Appear in parentheses preceded by a space, e.g., "as described previously (1, 2)"; "several research groups (4–10) have found."

☑ No superscript, boldface, italics, etc.

Figure and table callouts

☑ Figures and tables are called out in numerical order.

☑ "Figure," "Table," "Supplemental Figure," "Supplemental Table," etc., are spelled out.

☑ Callouts appear in parentheses (no boldface or italics) preceded by a space, unless grammatically part of the sentence: "the levels increased (Figure 5A)"; "data shown in Table 2."

☑ Parts are called out as, e.g., "Figure 1A," "Figure 2, A and B," "Figure 3, B–D."

Manuscript preparation and required reporting

☑ Title page

☑ Manuscript title

☑ Clear, concise, and limited to 15 words, including conjunctions.

☑ Refers to the relevant disease or disease model studied.

☑ No subtitles, colons, periods, or nonstandard abbreviations.

☑ Authors and affiliations

☑ Author names are provided in full (for example, "Benita J. Sjögren") and in the appropriate order.

☑ No titles, honorifics, degrees, or certifications.

☑ Affiliations correspond to the period when the work was performed.

☑ For authors whose affiliation has changed since completion of the work, specify the present affiliation and location below the numbered list.

☑ Affiliation footnotes are assigned consecutively using superscripted numbers (1, 2, 3, etc.).

☑ Affiliations include departments, institutions, city, state (if applicable), and country (but not mailing addresses or zip/regional codes).

☑ Corresponding author's complete name, address, telephone number (including country code if applicable), and email address.

☐ Consortium/study groups shown as authors (e.g., CARDIoGRAM Consortium)

- ☐ Unless the members of the group appear as authors, each individual member and their affiliation are listed in the supplemental material, under the heading Supplemental Acknowledgments.
- ☐ The following sentence appears in Acknowledgments: "See Supplemental Acknowledgments for details on {name of consortium}."

☑ Conflict-of-interest statement

☑ A statement consistent with the Journal's conflict-of-interest policy is included; if no author has a conflict, state the following: "The authors have declared that no conflict of interest exists."

☑ If patents are involved, the patent or patent application number(s) are provided and the names of the associated authors specified.

Created August 2021, updated June 2022

☐ Abstract

☐ Structured format with the sections Background, Methods, Results, Conclusion, Trial registration, Funding.
☐ Maximum 250 words.
☐ No references.
☐ All nonstandard abbreviations are defined at first use.

Main text (presented in the following order)

☐ Introduction

☐ Results

☐ Discussion

☐ Methods

☐ Demographic reporting (see details here)
  ☐ Reporting on race and ethnicity adheres to NIH guidelines or other applicable authoritative standards.
  ☐ Descriptors for any demographic identities are clear, unbiased, and up-to-date.
  ☐ Data for any demographic variable are inclusive; if any information is unavailable or incomplete, an explanation is provided.
  ☐ Specify whether the participants or investigators made the classifications; and whether the options were defined by the investigators or participants.
☐ Complete manufacturer name (omit location) is provided for each proprietary item used.
☐ For animal models, precise genotype, strain, number of backcrosses, sex, age, and source are specifed.
☐ Antibodies: Commercial — source and catalog/clone number are specified for each; custom — generation of antibodies is described (or an appropriate reference is cited).
☐ Source of all cell lines used is indicated.
☐ Data sets for gene expression microarrays, SNP arrays, and high-throughput sequencing studies are deposited in a public repository, and accession number(s) provided in Methods in the main text (for publication, data must be publicly available).
☐ Statistics
  ☐ Section appears near the end of Methods (before "Study approval").
  ☐ The *P* value used to determine significance of differences is specified; e.g., "A *P* value less than 0.05 was considered significant."
  ☐ Analysis appropriately corrects for multiple comparisons (more than 2 groups) and for repeated measures (multiple measurements within subjects).
  ☐ If samples were excluded, a statement describes inclusion/exclusion criteria.
☐ Study approval
  ☐ Stand-alone paragraph at the end of Methods.
  ☐ Declaration of approval of human and/or animal studies, specifying the official name and location of the applicable institutional review board(s).
  ☐ For human studies, a statement indicates receipt of written informed consent from participants and/or their parents/guardians.
  ☐ For use of photographs of participants, a separate statement of written informed consent is included.

☐ Author contributions

☐ Contribution of each author (identified by initials) is specified; e.g., designing research studies, conducting experiments, acquiring data, analyzing data, providing reagents, writing the manuscript. Multiple contributions may be listed for a single individual, and more than one individual may be associated with a single contribution.
☐ Grammatically complete sentences are used.
☐ For manuscripts with 2 or more co–first authors, the method used to assign authorship order among these authors is stated.

☐ Acknowledgments

☐ States sources of support in the form of grants, equipment, or drugs.
☐ Grant numbers are provided as applicable.
☐ Other acknowledgments, such as of colleagues for advice, are included as appropriate.

☐ References

☐ Prepared according to How to prepare references for submission.

☐ Figure legends

☐ Maximum 300 words.
☐ Each begins with a stand-alone title, irrespective of the individual parts.
☐ Figure parts are called out in boldface: (**A**), (**B**–**D**), (**C** and **E**).
☐ Symbols and abbreviations introduced in figures are defined and used consistently throughout.
☐ Use of terms within the legends is consistent with that in the figures themselves.
☐ In each figure legend where appropriate, the statistical test(s) used are described.
☐ For each panel representing multiple experiments, the exact number of samples (*n*) is reported.
☐ For representative experiments, the number of times the experiment was conducted is reported.
☐ Error bars are defined either in Statistics or in the individual legends; e.g., "Data represent mean ± SEM."
☐ Variance around the mean and statistical analysis are not provided for figures representing fewer than 3 independent samples.
☐ For histological panels and insets, scale bars are defined or total original magnification is specified in the legends.

☐ Figures

☐ Prepared according to How to prepare figures for submission.
☐ For clinical trials, the appropriate flow diagram appears as a figure.
☐ Parts are labeled with capital letters: A, B, C, etc., with no designated subparts.
☐ Graphs of quantitative data are presented as either dot plots, with average and appropriate error bars indicated; or box-and-whisker plots, with values defined in the legend (bounds of the boxes, lines within the boxes, whiskers, and any outlying values). Dynamite plunger plots are not permitted.
☐ If lanes in a gel or blot image are spliced together into a composite image, the lanes are distinguished with a thin vertical dividing line (black on a gray background; or white on a black background). State in the legend that the lanes were run on the same gel but were noncontiguous.

☐ Tables

☐ Prepared in Word table format (not pasted in from another application).
☐ Self-contained and self-explanatory.
☐ Preceded by brief titles.
☐ Each table fits on a single page and is presented on its own page.
☐ Callouts to footnotes (designated with superscript capital letters) are assigned alphabetically row by row.
☐ No subparts or subsections (for example, Table 1A and Table 1B).
☐ Column headings in tables apply to all values throughout the column; a new row of column headings may not be introduced within a table.
☐ See "Methods" above for reporting on demographics.
